# Isoflurane Induced Malignant Hyperthermia in a Patient with Glucose 6-Phosphate Dehydrogenase Deficiency and Growth Hormone Abuse

**DOI:** 10.1155/2020/8888368

**Published:** 2020-08-06

**Authors:** Hojatolah Ravaei, Mohammad Javad Yavari Barhaghtalab, Vahid Salehi, Hossein Hejr

**Affiliations:** ^1^General Surgery Department, Shahid Beheshti Hospital, Yasuj University of Medical Sciences, Yasuj, Iran; ^2^Anesthesiology Department, Shahid Beheshti Hospital, Yasuj University of Medical Sciences, Yasuj, Iran

## Abstract

Malignant hyperthermia is a pharmacogenetic disorder in the regulation of calcium in skeletal muscles which is related to an uninhibited muscle hypermetabolic reaction to potent inhalation agents, the depolarizing muscle relaxant succinylcholine, and to stressors such as vigorous exercise and heat. MH is diagnosed by the clinical presentation of the disease and laboratory testing. There are a few previous studies working on if there is an association between the occurrence of malignant hyperthermia and the existence of glucose 6-phosphate dehydrogenase (G6PD) deficiency, and there was no report on growth hormone doping in the literature. So, our main goal was to show this rare case of malignant hyperthermia seen in a G6PD patient with growth hormone abuse who underwent surgery and to find if there is an association between G6PD deficiency, growth hormone abuse, and malignant hyperthermia. Our patient was a 17-year-old boy with right lower quadrant abdominal pain and tenderness who underwent appendectomy. At the end of the operation, the patient developed with an increased heart rate (sinus tachycardia), increased body temperature and end-tidal carbon dioxide (ETCO2) level, masseter muscle rigidity, and then, generalized body rigidity, so the malignant hyperthermia susceptibility was considered. The patient was managed by cooling down the patient and the administration of dantrolene. We could hypothesize that malignant hyperthermia might be associated with G6PD deficiency as a triggering factor, but has no association with recombinant human growth hormone (rhGH) abuse. Another main lesson which this study tells us is to make a careful and proper history taking before going on an operation for preoperative evaluation and identification of patients with any form of suspicious drug abuse in order not to receive volatile inhalational agents and, also, performing some preventive measures including avoidance of heat extremes and restricting athletic activity in a patient with a history of malignant hyperthermia, and if the malignant hyperthermia susceptibility is suspected, urgent management should be carried out. As the association between G6PD deficiency, human growth hormone abuse, and malignant hyperthermia has remained unclear up-to-date, further potent studies are seriously needed in the future.

## 1. Background

Malignant hyperthermia (MH) is a pharmacogenetic disorder in the regulation of calcium in skeletal muscles which is related to an uninhibited muscle hypermetabolic reaction to potent inhalation agents (such as halothane, isoflurane, sevoflurane, and desflurane), the depolarizing muscle relaxant succinylcholine, and to stressors such as vigorous exercise and heat (environmental temperature). Another reason related to the development of MH is the genetic background and family history [1–3].

The frequency of MH is from 1 : 10000 to 1 : 250000 anesthesia. It occurs commonly at the first contact with anesthetic agents and affects men and young people more than women and elderly people. The mean age at the time of diagnosis is about 18.3 years [1].

Malignant hyperthermia may happen throughout the anesthesia, early after the surgery and up to an hour after the stoppage of volatile inhalational agents [4]. It may cause acidosis, hyperthermia, tachycardia, hypercapnia, tachypnea, increased oxygen consumption, masseter muscle, generalized body rigidity, compartment syndrome and rhabdomyolysis, increase in the serum creatine phosphokinase (CPK) level, hyperkalemia with an increased risk for cardiac arrhythmia, and myoglobinuria with a risk for renal failure [1, 2].

The first manifestations of MH in almost all the cases are tachycardia and an increased end-tidal carbon dioxide (ETCO2) level regardless of increased minute ventilation in the operating room [1, 2]. An early indicator of MH is muscle rigidity which can be seen in masseter muscle rigidity [4], and this is defined as the difficulty in manual mouth opening without temporomandibular dysfunction [5].

There are many reports studying various disorders and triggering agents associated with the occurrence of MH [1], but there are only a few previous studies working on if there is any association between the occurrence of malignant hyperthermia and existence of glucose 6-phosphate dehydrogenase (G6PD) deficiency in the literature [6, 7]. In addition, there were no previous reports on any association between MH and human growth hormone doping or abuse. So, our main goal was to show this rare case of malignant hyperthermia seen in a G6PD deficient patient with growth hormone doping who underwent surgery and to find if there is an association between G6PD deficiency, growth hormone doping or abuse, and MH.

## 2. Case Presentation

A 17-year-old boy who was 174-cm tall and weighed 65 kg admitted to the hospital due to abdominal pain since one day before admission. The pain was in the periumbilical area and right lower quadrant of the abdomen and started after an excessive exercise (boxing). The patient had no nausea, vomiting, but anorexia.

The patient was also a known case of G6PD deficiency since birth time. The G6PD deficiency test was conducted, and the result was deficient and confirmed the diagnosis. He had no hemolytic crisis, jaundice, and blood transfusion up-to-date. At first, in the hospital, before and after the operation, the patient and his family members did not give an appropriate history of growth hormone abuse, but gave a history of using growth hormone for growth problems and short stature under an endocrinologist follow-up from childhood; but after a precise history taking, we found that the patient had started illegally to use recombinant human growth hormone (a five mg injection under the skin) for bodybuilding and strength advantage from two month prior to surgery once a day at bed time (Norditropin FlexPro (somatropine) subcutaneous solution (5 mg/1.5 mL)). The patient had no delayed bone age, but had normal velocity growth. The patient had visited an endocrinologist 15 days prior to his admission to the hospital and was sent for a health check-up and also checking for insulin-like growth factor 1 (IGF 1) and a thyroid function test (TFT). TFT showed normal range T3, T4, and TSH as 1.51 nmol/ml, 9.1 g/dl, and 1.82 mIU/ml, retrospectively, and IGF 1 was 180.6 ng/ml (reference interval 75–850).

The patient had left wrist fracture 3 years before the current hospital admission, but no prior surgeries were found. Moreover, his grandparents and father received general anesthesia without recognized complications.

The patient's physical exam was normal except for right lower quadrant (RLQ) abdominal tenderness, rebound tenderness, and positive Rovsing's sign without any evidence of fever. Laboratory studies were unremarkable, and there was no evidence of active hemolysis. His hemoglobin (Hb) level was 14.1 g/dl; however, other hemolytic laboratory parameters were not evaluated because there was no history of G6PD deficiency-related sequelae such as hemolysis, jaundice, or blood transfusion. The patient was sent to the operation room with the impression of acute appendicitis for open appendectomy.

In the operation room, the patient's vital signs immediately before induction of anesthesia were as follows: pulse rate = 86 beat/minutes, blood pressure = 120/80 mm Hg, respiratory rate = 12 breaths/minutes, and axillary temperature = 36.7 degrees Celsius. In the supine position, under continuous electrocardiography, pulse oximetry, capnography, monitoring ETCO2, blood pressure monitoring, and temperature measurement (axillary), the patient underwent 3 min of denitrogenation with 100% oxygen through a facemask. Anesthesia was induced using midazolam (2 mg), thiopental sodium (200 mg), and isoflurane in oxygen using the Bain circuit. After confirming the loss of consciousness, atracurium (30 mg) and fentanyl (25 mcg) were administered, and then, endotracheal intubation was performed using a 7.5 mm tube (internal diameter) with an inflatable cuff. After applying the ventilator, ETCO2 was 34 mmHg. The patient received cefazoline 1 gr IV for one dose for antibiotic prophylaxis, and the operation started. Anesthesia was maintained with isoflurane in oxygen during operation.

At the end of the operation, about 45 minutes after starting the induction of anesthesia and starting surgery, while the patient was still intubated and going to be awake and having his own spontaneous respiration, he developed with (in order of occurrence) an increased heart rate to 124 beat/minutes (sinus tachycardia), increased body (axillary) temperature to about 41.7 degrees Celsius, gasping like respiration, increased ETCO2 to 153 mmHg, MMR, and then generalized body rigidity, so the malignant hyperthermia susceptibility was considered. Blood pressure was mildly elevated and was 140/80 mmHg. The color and volume of the perioperative urine was normal. Immediately, inhalational anesthetics were discontinued, and the Bain circuit and machine were exchanged to be replaced with an uncontaminated one; next, dantrolene rapid intravenous (IV) bolus (2.5 mg/kg) was given, and one vial of sodium bicarbonate was infused. An internal Foley catheter and nasogastric tube were inserted, and the bladder and the stomach were irrigated with cold saline, and the patient was hydrated with 1000 cc cold crystalloids for two times to reach the urine output of at least 2 cc/kg/hour. The patient was further cooled with a wet sponge applied to the surface of the body. About 30 minutes after administration of dantrolene and cooling measures, the heart rate was dramatically decreased to 80 beats/minutes, ETCO2 decreased from 153 to 35 mm Hg, and temperature decreased to 38.5°C (axillary), and cooling measures were stopped. The patient could not be extubated due to a low level of consciousness at the end of the operation, and was sent intubated to the surgical intensive care unit (ICU). In the ICU, dantrolene was continued at 1.0 mg/kg every 6 hours for 24 hours.

The patient had been in the hospital for twelve days; the first three nights were in the ICU for stabilization and, then, till the discharge day in the surgical ward. The patient was under close observation, and continuous monitoring of heart, blood pressure, temperature, and urine output was performed while checking for arterial blood gases (PH, partial pressure of carbon dioxide (PCO2), HCO_3_^−^ and base excess in the extracellular fluid compartment (BEecf)), white blood cell count (WBC), Hb, platelet count, liver enzymes as aspartate aminotransferase (AST), alanine aminotransferase (ALT), total and direct bilirubin, alkaline phosphatase (AlK–P), albumin (Alb), blood urea nitrogen (BUN), creatinine (Cr), blood sugar (BS), electrolytes (Na^+^ and K^+^), calcium (Ca^2+^), CPK, creatine kinase-muscle/brain (CKMB), and lactate dehydrogenase (LDH) (Tables 1 and 2).

In this case, the urine output did not decrease, the urine color did not change, and urine analysis was considered to be normal. Reticulocyte and corrected reticulocyte count were 1.5% and 1%, respectively. A peripheral blood smear was taken and showed no fragmented red blood cells (RBCs), but mild leukocytosis (neutrophilia). Direct Combs and indirect Combs tests were both negative. Viral markers as hepatitis B surface (HBS) antigen-antibody, hepatitis C virus (HCV) antibody, and human immune-deficiency virus (HIV) antibody were negative by the enzyme-linked immunosorbent assay (ELISA) technique. C-reactive protein (CRP) and uric acid were checked and were in the normal range (0.7 and 3.3 mg/dl retrospectively). Troponin I was checked and was positive, so it was titrated and was 41.5 ng/l, and echocardiography was performed which revealed a normal ventricular function without residual pulmonary valve stenosis or pulmonary regurgitation. The antinuclear antibody (ANA) titer was 0.289 and was negative. Ceruloplasmin was 0.398 g/l and was in the normal range. Serum sodium, magnesium (Mg), prothrombin time (PT), partial thromboplastin time (PTT), the international normalized ratio (INR), hemoglobin (Hb), platelet (Plt), blood urea nitrogen (BUN), and creatinine (Cr) were all in the normal range.

## 3. Discussion

In the pathophysiology of the MH, the triggering elements (inhalation agents and the depolarizing muscle relaxant succinylcholine) may cause the calcium stores to be released from the sarcoplasmic reticulum, uncontrolled entry of calcium from the myoplasm, activation of biochemical routes related to muscle activation causing the contracture of skeletal muscles, glycogenolysis, and increased cellular metabolism, all of which resulting in the production of heat and increased lactate level and acidosis [1, 2]. The mechanism of dantrolene is to act as an antagonist, and it binds to a specific region in the ryanodine receptor 1 (RYR-1) channel. It reduces the uncontrolled release of intracellular calcium and should be available anywhere when general anesthesia is performed [1, 8].

Clinical examination or laboratory testing is vital for the diagnosis of MH. Unexplained elevation of the ETCO2 level, muscle rigidity, tachycardia, acidosis, hyperthermia, and hyperkalemia are the main diagnostic features of MH [1]. In this case, the first sign was tachycardia, the 2^nd^ was increased temperature, and the 3^rd^ was an increased ETCO2 level which accompanied by masseter muscle and generalized body rigidity. The patient developed with severe metabolic and respiratory acidosis, and this was due to uncontrolled hypermetabolism and rapid consumption of energy stores and adenosine triphosphate (ATP) [1].

**Figure 1:**
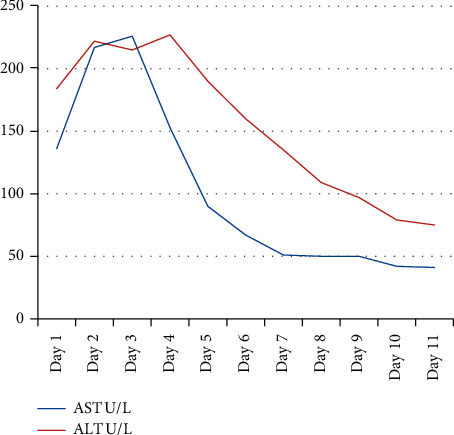
Decreasing trend in AST and ALT.

**Figure 2:**
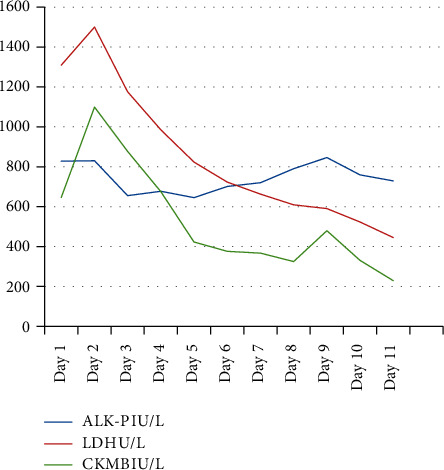
Decreasing trend in ALK-P, CKMB, and LDH.

**Figure 3:**
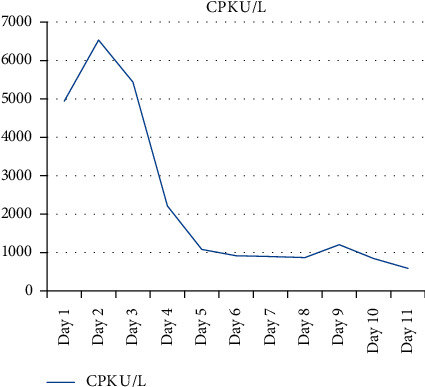
Decreasing trend in CPK.

An increased CPK level is the first indicator of MH susceptibility [1]. In our case, the diagnosis of rhabdomyolysis is confirmed by an increased level of muscle enzymes, CPK, AST, ALT, CKMB, and LDH levels. The decreasing trend of CPK, CKMB, LDH, and AlK-P is shown in Figures 1–3. There was no kidney injury or subsequent renal failure, so there were no complications due to rhabdomyolysis in our case study.

There is a clinical grading scale developed by Larach and colleagues through a Delphic study of experts [9]. Each of the manifestations of the syndrome is given a point according to its importance in differentiating MH from other differential diagnoses, and then points are summed to produce a raw score which then this summed score translates to a likelihood score range from 1 (score 0: “nearly never/questionable or improbable”) to 6 (score ≥50: “nearly certain”). The more the criteria a patient achieves, the more likely that an MH episode has occurred. To be qualified for the scoring system, just one hint in each process is needed to be present [1, 2]. Using this clinical grading scale in our patient resulted in score 86 (Table 3), so according to the likelihood score ≥50, MH susceptibility diagnosis would be “almost certain”.

However, clinical manifestations of MH happen during the administration of the triggering agent in most reported cases, and there are some reports in which the MH manifestations began following surgical intervention. The period between the administration of an anesthetic triggering agent and the start of the MH manifestations have a case to case variability, but the utmost latency period is indefinite [3]. In our case, MH manifestations were started at the end of the operation, but during the administration of the triggering agent and with a gap about 45 minutes from the starting point of triggering agent administration.

Some differential diagnosis of MH during anesthesia includes sepsis, thyroid storm, pheochromocytoma, and iatrogenic warming [1]. In our case study, sepsis, thyroid storm, and pheochromocytoma were ruled out due to a negative CRP, a normal TFT, and normal preoperation blood pressure, retrospectively. Unexplained increased ETCO2 could be due to hyperthermia due to sepsis, iatrogenic overheating, respirator valve malfunction, rebreathing, and faulty equipment [1]. These differential diagnoses were all ruled out because all the equipment and machine were checked before operation by the anesthesiologist technician; moreover, they were used successfully for the patients operated before the time of this operation. There are some differential diagnoses when encountering MH-like manifestations outside the operating room such as injection of ionic contrast agents into the cerebrospinal fluid, cocaine overdose, neuroleptic malignant syndrome (NMS), serotonin syndrome, and 3, 4-methylenedioxy-methamphetamine (MDMA) overdose [1].

Several factors may have contributed to the rapid rise in temperature, and malignant hyperthermia was seen in this patient. The most important cause is the administration of isoflurane, and this was like other case reports [10–12]. There is an association between MH, exertional heat injuries, and exertional rhabdomyolysis [13]. In our study, vigorous exercise (boxing) by the patient on the day before operation might trigger this effect. Also, the generation of heat might have been increased by the body movement and shivering associated with the steepest part of the rise in temperature. Environmental (operation room) temperature might be another trigger factor for inducing hyperthermia because it was summer at the time of operation, and air conditioners did not work well (room temperature was 27.2°C). Heat loss may have been limited by the excessive surgical draping which covered nearly all surfaces during operation.

G6PD deficiency is the most common genetic enzymatic disorder of red blood cells in humans and causes hemolytic anemia after exposure to oxidative stresses such as certain drugs, infections, metabolic conditions, and ingestion of fava beans. It is expected that there are about 400 million people with this disorder worldwide. Important concerns in the management of anesthesia in these patients include avoiding oxidative stresses and monitoring for hypercapnia due to acidosis and hemolysis [14–16].

Younker et al. reported a 22-month-old baby with G6PD deficiency who developed with MH. They concluded that decreased major antioxidant system activity may cause susceptibility to MH [6]. It has been proposed that oxidative damage, especially in membranes, may be the reason for the occurrence of MH [17]. The major antioxidant systems are the hexose monophosphate (also called the phosphogluconate pathway and the hexose monophosphate shunt) and the glutathione pathways. In animal models such as swine with MH, a deficiency has been demonstrated for Se-dependent glutathione peroxidase (Se-GPx), or G6PD. In human models with MH, a deficiency has been found in Se-GPx, glutathione, glutathione reductase, or 6-phosphogluconate dehydrogenase [7]. So, it shows that the antioxidant deficit may be a prime reason which causes protective mechanisms not to be active against the occurrence of MH and may be a prerequisite for MH [7, 17].

G6PD is the first enzyme of the pentose phosphate pathway that converts *α*-D-glucose-6-phosphate into D-glucono-1,5-lactone-6-phosphate and is involved in the generation of nicotinamide adenine dinucleotide phosphate (NADPH). As erythrocytes lack the citric acid cycle, the pentose phosphate shunt is the only source of NADPH. NADPH is required for the generation of reduced glutathione, which is important for the protection against oxidative damage [18].

Altikat et al. found that isoflurane, sevoflurane, diazepam, and midazolam had an inhibitory effect on G6PD activity in vitro [19]. Cho et al. found that thiopental sodium, sevoflurane, fentanyl, pethidine, and rocuronium were safe in their G6PD deficient patient [15].

Although there are a few research studies conducted to study the association of G6PD deficiency and MH occurrence and the effects of anesthetic agents on the G6PD deficient patients are unclear, it seems that G6PD deficiency could be a trigger for developing MH in our case. As MH is a hazardous risk factor for susceptible persons undergoing general anesthesia using volatile agents and there is no evidence-based agreement about the use of anesthetic agents in MH patients, oxidative stresses should be avoided and hemolysis should be detected and monitored as key elements in a perfect anesthesia in such patients.

Growth hormone is a naturally occurring peptide hormone produced by the anterior pituitary gland. It plays a major role in maintaining body composition, physical well-being, GH performance, and cardiovascular health in human. These properties have led to its abuse by athletes wishing to improve their muscular performance. Despite its apparent widespread use, until recently, there was little scientific evidence to support its use as a performance-enhancing agent [20]. According to the association between rhGH doping and hyperthermia, we could not find any previous relevant publish reports up-to-date, so we could hypothesize that MH might not be associated with rhGH doping or abuse.

## 4. Conclusions

We could hypothesize that the malignant hyperthermia might be associated with the G6PD deficiency as a triggering factor, but has no association with rhGH doping. Another main lesson which this study tells us is to make a careful and proper history taking before going on an operation for preoperative evaluation and identification of patients with any form of suspicious drug abuse in order not to receive volatile inhalational agents and, also, taking some preventive measures including avoidance of heat extremes and restricting athletic activity in a patient with a history of malignant hyperthermia, and if the malignant hyperthermia susceptibility is suspected, urgent management should be performed. As the association between G6PD deficiency, human growth hormone abuse, and malignant hyperthermia has remained unclear up-to-date, further potent studies (especially animal models) are seriously needed in the future.

## Figures and Tables

**Table 1:** MH-associated laboratory finding in the 1^st^ day of hospitalization.

	Normal range	Before operation	In the end of operation	30 minutes after the end of operation	1 hour after the end of operation	2 hours after the end of operation	6 hours after the operation
PH	7.35–7.45		6.81	7.18	7.33	7.38	7.47
PCO2	35–45 mmHg		>150	84	44.8	42.3	31.6
HCO_3_^−^	22–28 milliequivalents per liter (mEq/L)		4.5	31.4	24.1	25	23.4
BEecf	0 ± 4 mEq/L		−12.4	−2.8	−1.9	−0.3	−0.4
K^+^	3.6–5.2 millimoles per liter (mmol/L)			6	5.6		
Ca	8.6–10.3 mg/dL			15.4	7.9		8.2
WBC	4,500–11,000 WBCs per microliter	7000			19400		
CKMB	5–25 IU/L				543		646
LDH	140–280 units per liter (U/L)						1309
CPK	39–308 U/L in males						4942
AST	7–56 units per liter (U/L)				136		
ALT	7–56 units per liter (U/L)				184		
Alk-P	44–147 international units per liter (IU/L)				828		

**Table 2:** MH-associated laboratory finding from the 2^nd^ day to 11^th^ day of admission (one day before discharge).

	Day 2	Day 3	Day 4	Day 5	Day 6	Day 7	Day 8	Day 9	Day 10	Day 11
PH	7.40	7.41	7.44	7.38	7.37	7.36				
PCO2	38.6	37.6	37.1	41	40.2	41				
HCO3	24.3	24.3	26.4	24.6	23.6	24.3				
K^+^	4	4	3.6	4.2	4.4	4.7				
Ca^2+^	9.1	9.2	10	9.1	10.1	12				
WBC	13300	12100	12500	6700	6600	6900				
CKMB	1099	877	675	423	376	367	325	479	331	229
LDH	1500	1176	985	823	723	663	609	590	523	445
CPK	6532	5437	2213	1080	915	897	867	1203	843	588
AST	217	226	153	90	67	51	50	50	42	41
ALT	222	215	227	190	160	135	109	97	79	75
Alk-P	830	655	677	645	701	720	790	846	759	729

**Table 3:** Clinical grading scale in our case.

Process	Clinical elements in our case	Score
Muscle rigidity	Generalized body and masseter muscle rigidity (+)	15
Muscle breakdown	Elevated CPK > 10,000 IU after anesthesia without succinylcholine (negative), cola-colored urine (negative), excess myoglobin in urine or serum (not checked), and plasma [*K*+] > 6 mEq/L (in absence of renal failure) (negative)	3
Temperature increase	Rapidly increasing temperature, *T* > 38.8°C (+)	15
Cardiac involvement	Unexplained sinus tachycardia (+)	3
Respiratory acidosis	PETCO2 > 55 mmHg and arterial PaCO2 > 60 mmHg with appropriately controlled ventilation (+), inappropriate hypercapnia, and tachypnea (+),	15
Metabolic acidosis	Base deficit > 8 mEq/L (+), pH < 7.25 (+)	20
Reaction to dantrolene	Rapid reversal of malignant hyperthermia signs of metabolic and/or respiratory acidosis with intravenous dantrolene (+)	5
Elevated CPK	Elevated resting serum CPK (+)	10
Family history	Autosomal dominant inheritance (negative)	No point (0) out of 15
		Total score = 86

## Data Availability

The data used to support the findings of this study are available from the corresponding author upon request.

## Abbreviations

MH: Malignant hyperthermia
G6PD: Glucose 6-phosphate dehydrogenase
rhGH: Recombinant human growth hormone
CPK: Creatine phosphokinase
RYR-1: Ryanodine receptor 1
IGF1: Insulin-like growth factor 1
ETCO2: End-tidal carbon dioxide
BEecf: Base excess in the extracellular fluid compartment
WBC: White blood cell count
Hb: Hemoglobin
AST: Aspartate aminotransferase
ALT: Alanine aminotransferase
AlK-P: Alkaline phosphatase
Alb: Albumin
BUN: Blood urea nitrogen
Cr: Creatinine
BS: Blood sugar
CKMB: Creatine kinase-muscle/brain
LDH: Lactate dehydrogenase
RBCs: Red blood cells
HBS: Hepatitis B surface antigen antibody
HCV: Hepatitis C virus antibody
HIV: Human immune-deficiency virus antibody
ELISA: Enzyme-linked immunosorbent assay
CRP: C-reactive protein
TFT: Thyroid function test
MDMA: 3, 4‐methylenedioxy-methamphetamine
G/dl: Gram/deciliter
U/L: Units per liter
WHO: World Health Organization
Se‐GPx: Se‐dependent glutathione peroxidase
NADPH: Nicotinamide adenine dinucleotide phosphate
CO2: Carbon dioxide
LDL: Low density lipoprotein.
ANA: Antinuclear antibody
